# Neurocognitive mechanisms underlying value-based decision-making: from core values to economic value

**DOI:** 10.3389/fnhum.2013.00398

**Published:** 2013-07-24

**Authors:** Tobias Brosch, David Sander

**Affiliations:** Department of Psychology, Swiss Center for Affective Sciences, University of GenevaGeneva, Switzerland

**Keywords:** decision making, core values, neuroimaging, value-based decision making, value

## Abstract

Value plays a central role in practically every aspect of human life that requires a decision: whether we choose between different consumer goods, whether we decide which person we marry or which political candidate gets our vote, we choose the option that has more value to us. Over the last decade, neuroeconomic research has mapped the neural substrates of *economic value*, revealing that activation in brain regions such as ventromedial prefrontal cortex (VMPFC), ventral striatum or posterior cingulate cortex reflects how much an individual values an option and which of several options he/she will choose. However, while great progress has been made exploring the mechanisms underlying concrete decisions, neuroeconomic research has been less concerned with the questions of why people value what they value, and why different people value different things. Social psychologists and sociologists have long been interested in *core values*, motivational constructs that are intrinsically linked to the self-schema and are used to guide actions and decisions across different situations and different time points. Core value may thus be an important determinant of individual differences in economic value computation and decision-making. Based on a review of recent neuroimaging studies investigating the neural representation of core values and their interactions with neural systems representing economic value, we outline a common framework that integrates the core value concept and neuroeconomic research on value-based decision-making.

“All sciences are now under the obligation to prepare the ground for the future task of the philosopher, which is to solve the problem of value, to determine the true hierarchy of values”

- Friedrich Nietzsche

Value is arguably one of the most central concepts governing human life, as it is involved in practically every aspect that requires a decision: whether we choose between different consumer goods, whether we decide which person we marry or which political candidate gets our vote, whether we ask ourselves if something is beautiful, morally right, or sacred, value plays a crucial role. Value reflects the importance that something holds for us, what doesn't have any value is of no interest. Consistent with the central role of value in our lives, ever since Plato scholars have been trying to understand what value is and where it comes from. Today, the investigation of value is central to many disciplines studying human feeling, thinking and behavior, such as philosophy, psychology, sociology, economics, or neuroscience (Brosch and Sander, forthcoming).

Interestingly, the different disciplines are all focusing on somewhat different aspects and conceptualizations of value. According to the Oxford Dictionary of English, the word value in its broadest sense refers to the “importance, worth, or usefulness of something.” This general definition is followed by several subdefinitions, the first of which describes value as “the material or monetary worth of something.” This definition reflects how economists and neuroscientists think about value: A “common currency” that people use to compare different types of goods or experiences on the same scale when deciding between several options. *Economic value* is related to the amount of reward that a person expects to obtain from the choice. Over the last decade, neuroeconomic research has substantially increased our knowledge of the neural substrates representing value and the neurocognitive mechanisms underlying decision-making (Schultz, 2006; Rangel et al., 2008; Kable and Glimcher, 2009; Grabenhorst and Rolls, 2011; Padoa-Schioppa, 2011; Rushworth et al., 2011; Lee et al., 2012). While making great progress exploring the mechanisms underlying concrete decisions, neuroeconomists have put less emphasis on the questions of why people value what they value, and why different people value different things. This aspect is addressed in the second subdefinition of value in the Oxford Dictionary of English, “principles or standards of behavior, one's judgment of what is important in life.” This definition resonates with how social psychologists and sociologists think about value: A broad motivational construct at the core of the self-image that guides choices and behaviors across situations, often framed as a shared belief about ideal objectives (Rohan, 2000). Value research in social psychology and sociology focuses on the role of universals and individual and cultural differences in *core value* systems, and has shown that people in many different cultures use and recognize the same set of core values, but may differ in terms of their relative value priorities.

Thus, research on economic value has produced many insights into the neurocognitive mechanisms that drive decisions in concrete situations, whereas research on core value allows explaining interindividual differences in decision situations as well as intraindividual consistency across decisions over time. Whereas these different facets of the value concept so far have been investigated more or less in isolation from each other, we feel that an integration of the two perspectives would be extremely useful. In this contribution we review (a) neuroeconomic research delineating the neurocognitive mechanisms underlying economic value computations and (b) social psychological and sociological research concerning the universal structure of core values and the role of individual core value differences in decisions and behaviors. We then propose a common framework that aims at integrating the core value concept into a neuroscience of decision-making, and support our idea by a review of recent neuroimaging studies investigating the neural representation of core values and their potential interactions with neural mechanisms underlying value computation and decision-making.

## Economic value: a common currency for decision-making

In economic and neuroeconomic theory, value is conceptualized as a measure of the benefit that people can gain from choosing an option. When having to decide between several options, the person will compute the value of each option, and then choose the one with the highest value. The value of an option is derived from a person's behavior, i.e., the observable choices of the individual: If a person chooses option A over option B, it is inferred that option A has higher value. At the computational level, value depends on how much reward a person expects to receive from choosing an option, e.g., from eating a piece of chocolate or from receiving an amount of money. The notion of value as a common currency (Samuelson, 1947) is central to many economic theories of decision-making, as it allows to conceptualize how people can compare and choose between different types of rewarding objects. To illustrate the problem, whereas the decision between different amounts of the same rewarding object is relatively straightforward (e.g., “Would you prefer one piece of chocolate or two pieces?” or “Would you prefer $5 or $10?”), choosing between two objects with several different reward-related attributes is more complex (e.g., “Would you prefer a piece of chocolate or a salad?”), as different dimensions (e.g., considerations pertaining to taste and to health, respectively) need to be taken into account and weighed against each other. In these cases, a common value currency allows integrating and combining the different dimensions into one representation that can be used as a basis for individual decisions.

Over the last decade, the brain network representing economic value has been delineated using neuroimaging methods (Padoa-Schioppa and Assad, 2006; Schultz, 2006; Kable and Glimcher, 2009; Grabenhorst and Rolls, 2011; Padoa-Schioppa, 2011; Rushworth et al., 2011; Lee et al., 2012; Levy and Glimcher, 2012) as well as single neuron recordings (in primates, Platt and Glimcher, 1999; Tremblay and Schultz, 1999). In a typical neuroimaging experiment, participants view different stimuli (for example different consumer objects) and are asked to choose one of them (or to indicate how much they like each option). The individual choices (or preferences) are then used to derive a measure of economic value, which is used as a parametric regressor to identify brain regions that show systematic activation changes as a function of the value of the presented objects. A large number of converging studies have identified a network of brain areas representing subjective economic value for many different types of rewarding stimuli, consisting of ventromedial prefrontal cortex/orbitofrontal cortex (VMFPC/OFC), ventral striatum, posterior cingulate cortex, amygdala, insula and posterior parietal cortex (see, e.g., Kawabata and Zeki, 2004; O'Doherty, 2004; Kim et al., 2011; Levy and Glimcher, 2012).

Studies comparing neural activation to different classes of rewarding stimuli in the same subjects (e.g., to food, consumer goods, money, or social reputation gains) have observed overlapping activations in VMPFC/OFC, striatum, and insula, suggesting that these regions indeed represent a common currency for different types of rewarding stimuli that allows comparing and deciding between objects with very different properties (Izuma et al., 2008; Chib et al., 2009; Grabenhorst et al., 2010; Kim et al., 2011; Lin et al., 2012). This neural system representing economic value can implement computations of considerable complexity, such as a cost-benefit analysis (when participants are choosing between options that imply both rewarding and punishing aspects) in interactions of VMPFC/OFC and insula (Talmi et al., 2009), and value discounting during delay of gratification (when participants are choosing between a smaller reward right now and a higher reward later) in VMPFC/OFC and ventral striatum (McClure et al., 2004).

Activation in this network should thus allow to infer preferences and to predict choices: When two different objects elicit neural activation of equal magnitude, the two objects should be equally desirable for a person. In contrast, when activation is increased toward one object compared to another, this object should be preferred. And indeed, measurements of brain activation in regions of this network allow predicting which of two items an individual prefers and choses, at least when the subjective value difference between the two items is fairly large (FitzGerald et al., 2009; Lebreton et al., 2009; Levy et al., 2011).

To sum up, neuroeconomic research has reliably identified a brain network representing economic value that allows predicting individual preferences and choices. However, whereas much progress has been made identifying the neurocognitive mechanisms underlying concrete choices, neuroeconomic research has mostly neglected questions such as why people choose (and thus value) what they choose, or why different people choose (and thus value) different things. At the proximal level, this question has been addressed by looking at the impact of individual reinforcement learning histories (see Lee et al., 2012, for a review) However, more research on the distal motivational principles that can predict decisions across situations is clearly needed. Moreover, neuroeconomic research is largely restricted to relatively simple decisions, such as choices between two consumer goods, and rarely investigates more complex decisions and life choices. Such issues are however addressed by researchers interested in core value, mainly from social psychology and sociology. In the following section, we will summarize some key concepts and findings from this field.

## Core value: a stable concept of what is desirable

Core value refers to stable motivational constructs or beliefs about desirable end states that transcend specific situations and guide the selection or evaluation of behaviors and events (Rohan, 2000). An individual's core values form an internal compass that people refer to when they are asked to explain and justify their preferences, decisions, or behaviors. For example, a person may frequently donate money to charitable causes and explain this behavior by their altruistic core values. Core values are thus instrumental in providing the individual with meaning in the world. They provide an organizational principle for an individual's self-schema (Roccas and Brewer, 2002), forming the core of one's identity (Hitlin, 2003).

Cross-cultural research has shown that certain core values are universal, meaning that people in many different cultures can recognize and use the same core values to describe their personal core value hierarchy (see Table 1; Schwartz, 1992).

**Table 1:** **The 10 universal core values and their conceptual definitions (Schwartz, 1992)**.

**Value**	**Conceptual definition**
Self-direction	Independent thought and action—choosing, creating, exploring
Stimulation	Excitement, novelty, and challenge in life
Hedonism	Pleasure and sensuous gratification for oneself
Achievement	Personal success through demonstrating competence according to social standards
Power	Social status and prestige, control or dominance over people and resources
Security	Safety, harmony, and stability of society, or relationships, and of self
Conformity	Restraint of actions, inclinations, and impulses likely to upset or harm others and violate social expectations or norms
Tradition	Respect, commitment, and acceptance of the customs and ideas that traditional culture or religion provides
Benevolence	Preservation and enhancement of the welfare of people with whom one is in frequent personal contact
Universalism	Understanding, appreciation, tolerance, and protection for the welfare of *all* people and for nature

**Figure 1:**
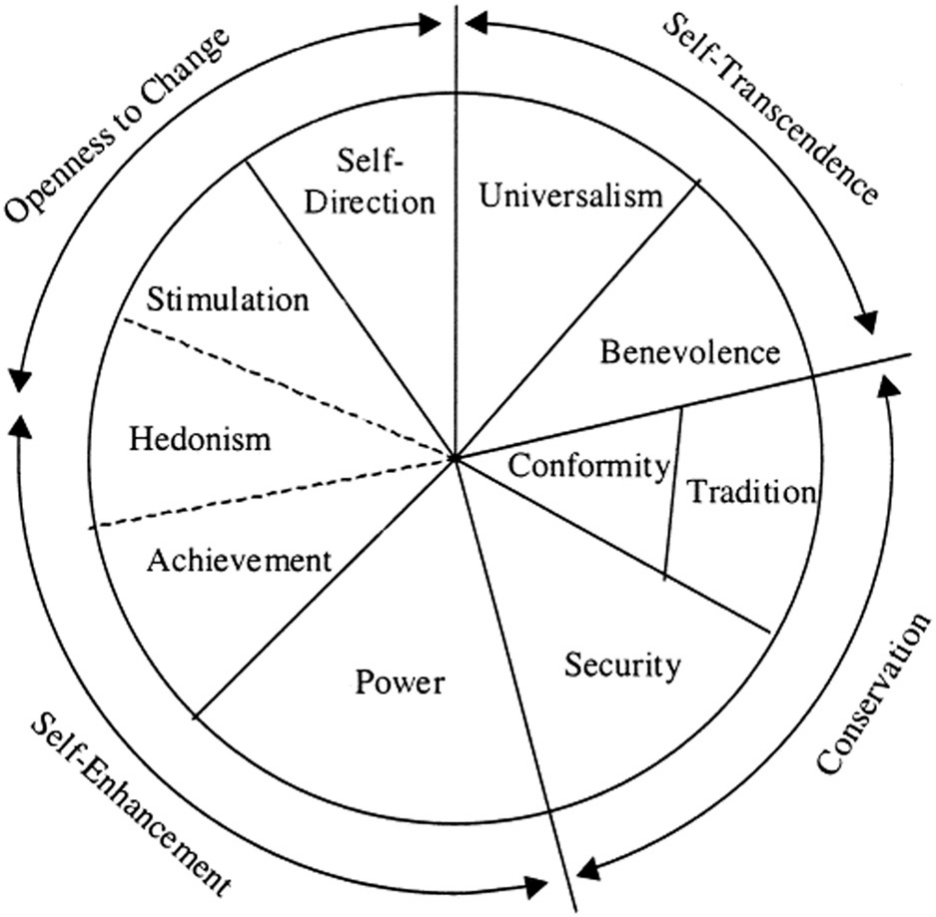
**Circumplex formed by the 10 universal core values (illustration reproduced with permission from Olver and Mooradian, 2003)**.

These 10 core values can be grouped in a circumplex where they form clusters organized along two core value dimensions, which reflect conflicts between opposing classes of human interests (see Figure 1). The first dimension is labeled “self-enhancement vs. self-transcendence,” and reflects the conflict between outcome maximization for the individual vs. outcome maximization for the social group. Individuals with highly self-interested core values emphasize power and achievement-related goals and choices, whereas individuals with self-transcending values emphasize universal and benevolent goals and choices. The second dimension is labeled “openness to change vs. conservation,” and reflects the conflict between following one's interests in uncertain directions vs. preserving the status quo embedded in existing relationships. Individuals with conservative values emphasize conformity, security, and tradition, whereas individuals with open-to-change values emphasize self-directive and stimulating goals and choices (Schwartz, 1992).

Importantly, core values are not only used to give orientation and stability to the self, but allow predicting individual differences in concrete decisions and behaviors. For example, a person emphasizing conservation-related values more frequently observes traditional customs on religious holidays than a person who does not hold these values in high esteem. A person who emphasizes self-transcending values more frequently uses environmentally friendly products than a person who emphasizes self-enhancing values (Bardi and Schwartz, 2003). Core value differences have furthermore been shown to be powerful predictors of voting behavior (Schwartz et al., 2010). Thus, the core value concept is a powerful construct that may explain why different people value different things and why different people choose differently in the same situation, and thus may be fruitfully combined with neuroeconomic research on value computation and decision-making.

However, so far not much research has attempted to investigate the neural mechanisms underlying the role of core value in decision-making. In a first attempt to integrate core value into current neuroimaging research, we aimed at identifying the neural regions involved in the representation of core value (Brosch et al., 2012). To this end, we showed our participants examples of behaviors that reflect different core values (e.g., “correcting injustice,” “respecting traditions”) and asked them to indicate on a scale from 1 to 4 how important the behavior (and thus the related core value) is for them (*core value condition*). In order to directly compare the neural regions representing core value to the regions representing economic value, these behaviors were intermixed with examples of potentially rewarding concrete activities (such as “eating an apple,” “playing tennis”), for which participants indicated (using the same scale from 1 to 4) how much they like performing this activity (*economic value condition*). The economic value condition activated the expected neuroeconomic value network, including regions such as VMPFC, posterior cingulate cortex, and posterior parietal cortex. In contrast, the core value condition led to increased activation in medial prefrontal cortex (MPFC) and in the dorsal striatum. MPFC has frequently been linked to processes involving self-reflection (Macrae et al., 2004; Northoff and Bermpohl, 2004; Mitchell et al., 2005; Lieberman, 2010), both when explicitly reflecting about one's self and when implicitly processing self-related information (Rameson et al., 2010), and has furthermore been shown to be activated when thinking about future goals, which are closely tied to one's core values (D'Argembeau et al., 2009). The observed activation of MPFC is thus consistent with the conceptualization of core value as an integral part of the self-schema (Hitlin, 2003). However, given that so far this is the only neuroimaging study linking core value to MPFC, it would be important to replicate this finding in future studies.

## From core values to economic value: a common framework for value-based decision-making

As outlined in the previous sections, economic value and core value both refer to evaluative representations that guide decisions and behaviors. They are however conceptualized at different levels of situational concreteness, with economic value referring to a common currency that operates in concrete choice situations, and core value referring to motivational constructs that guide choices and behaviors across many situations. Despite the conceptual similarities, there has not been much integration and cross-fertilization between the two research traditions. We suggest combining the two value concepts into a common framework for decision-making. In linking these two concepts, neuroeconomic research may be enriched by an elaborate and empirically validated concept that allows predicting and explaining individual differences in value-based decision-making. Furthermore, integrating the set of core values and the related behaviors into neuroeconomic research goes beyond the kind of choices that are usually investigated empirically, moving from simple choices between consumer goods to a more diverse and complex array of choices. In return, core value research may gain a deeper understanding of the underlying mechanisms by which core values impact on decisions and behaviors. In this context, several core value researchers have suggested that the effects of core value on decisions and behaviors are relatively indirect, being exerted by changing the beliefs and norms of the individual (Dietz et al., 2005) or by exploiting one's need for consistency between beliefs and actions (Rokeach, 1973).

Here we want to evaluate the possibility that, in addition to these indirect effects, a more direct connection links core value, economic value, decision-making and behavior. Our hypothesis is that individual differences in core value may be determinants of how much economic value is given to the different options in concrete choice situations. Thus, the behavioral effects of core value differences may—at least partly—be implemented by neural mechanisms underlying the computation of economic value. In what follows, we will review the relevant neuroimaging evidence against which our hypothesis can be evaluated. Whereas to our knowledge only two studies have so far directly addressed the impact of core values on neural activation (Brosch et al., 2011, 2012), a number of other neuroeconomic studies have investigated the neural correlates of a specific behavior that is relevant to the core value dimension of self-enhancement vs. self-transcendence: egoistic vs. altruistic behavior expressed by charitable donations. The first neuroimaging study to investigate the neural correlates of charitable donations (Moll et al., 2006) presented participants with a series of choices on whether to donate money to a charitable organization related to a major societal cause (such as children's rights, gender equality, or nuclear power). In other trials, participants received money for themselves. Results revealed increased activation of the striatum, a central part of the neural system representing economic value, both when participants received money for themselves and when they decided to donate for a good cause. In further research, the perceived value of charitable donations has been shown to be represented in VMPFC/OFC as well (Hare et al., 2010). Taken together, these findings suggest that receiving money and donating money are both rewarding experiences, as expressed by a shared anatomical system of value representation. These findings were extended by demonstrating that increased striatal responses to charitable money transfers also occur when the transfer is mandatory (similar to an income tax), but that the striatal response is even higher when people voluntarily decide to make a donation (Harbaugh et al., 2007). In another study, participants were matched into pairs and presented with a series of unequal monetary distributions, where one participant received a large monetary endowment and the other one nothing (Tricomi et al., 2010). Participant who had already received a lot of money in previous trials showed a stronger neural response in VMPFC/OFC and ventral striatum when they observed a money transfer to the other participant (who had previously received less money), compared to when they received money themselves, indicating that the neural value regions also represent value related to distributive fairness. Finally, in a study on moral dilemmas, participants were confronted with scenarios where they had to make decisions that sacrificed the lives of some people in order to save others. The expected “moral reward value” (i.e., the ratio of lives saved/lost) was tracked by VMPFC/OFC and ventral striatum, suggesting that decisions based on self-transcending values may involve the same neural systems that represent economic value (Shenhav and Greene, 2010). Taken together, these results suggest that the neural regions representing economic value are involved in decisions and behaviors that are related to core values.

But are individual differences in the activation of these regions related to actual differences in altruistic decisions and behaviors? In the taxation-donation study by Harbough and colleagues described above, participants who showed a stronger striatal response when receiving money for themselves opted less frequently to donate money to charity (Harbaugh et al., 2007). Furthermore, in a study looking at individual differences in preferences for distributive fairness, participants who generally choose equal distributions of money showed increased amygdala activation when confronted with very uneven distributions (Haruno and Frith, 2010). These two studies suggest that behavioral differences that are relevant to core values may indeed be driven by differences in activation of neuroeconomic value regions.

**Figure 2:**
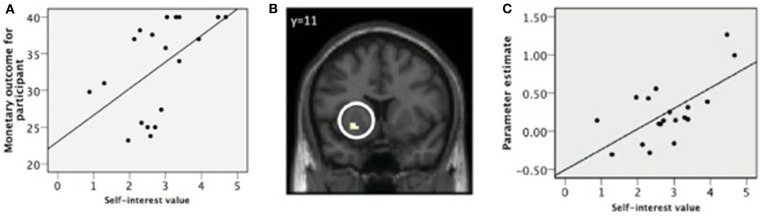
**Impact of self-centered core value hierarchies on neural regions representing economic value and on charitable behavior. (A)** Participants with a self-centered core value hierarchy kept more money for themselves instead of donating it to charity. **(B)** The same participants showed increased activation in the ventral striatum when receiving monetary rewards. **(C)** Correlation between self-interest value and parameter estimates for ventral striatum (reproduced with permission from Brosch et al., 2011).

As a final step in our argumentative chain, it remains to be shown that different neural activation patterns in economic value regions are actually related to individual differences in the core value hierarchy. To address this issue, we measured the core value hierarchies of individuals who participated in a donation task (Brosch et al., 2011). In some trials, participants could gain money for themselves, in other trials they decided whether they wanted to donate some of their money to charity. Analysis of the decisions made during the task showed that participants with self-centered core value hierarchies donated less money to charity, demonstrating that more self-interested core values are actually reflected in more selfish behavior (see Figure 2). At the neural level, all our participants showed increased activation of the striatum when receiving money. However, the activation was more pronounced for participants with a more self-centered core value hierarchy, suggesting that egoistic behavior is potentially more rewarding for participants with self-centered core values than for less self-centered participants.

Participants with self-centered core values furthermore showed a stronger neural response of the amygdala when having the opportunity to gain money for themselves, consistent with the suggestion that the amygdala acts as a relevance detector that is sensitive to the motivational salience of a stimulus given the current needs, goals and values of the organism (Davis and Whalen, 2001; Sander et al., 2003; Pessoa, 2010; Cunningham and Brosch, 2012).

Somewhat surprisingly, participants showed decreases in striatal activation when deciding to donate their money to charity, consistent with striatal deactivations observed during financial loss (Delgado et al., 2003), which is in contrast to studies reviewed above that reported increased striatal activation during altruistic donations (Moll et al., 2006; Harbaugh et al., 2007). The difference between our results and the results by Harbaugh et al. (2007) and Moll et al. (2006) may be due to contextual or methodological differences. For example, in the study by Moll and colleagues, participants were confronted with a different charitable organization in each trial, which included also organizations whose goals were not endorsed by the participants, whereas in our study, participants always donated to the same charitable organization that was chosen by the participant in advance. Furthermore, in the study by Harbaugh and colleagues, the monetary payoff to the charity was not correlated with the financial loss by the participant (i.e., the experiment contained trials where the participant lost USD 45, but the charity only received USD 15, as well as trials where the participant lost USD 45 and the charity received all of it). The striatal response reflects increased activation to increased monetary payoff to the charity; this analysis is thus not sensitive to the effects of the financial loss by the participant.

Taken together, striatal activation differences have been shown to be linked to behaviors reflecting self-interested as well as self-transcendent core values. Furthermore, our results point to an additional neurocognitive process involved in self-transcendent behavior that involves social cognition mechanisms: In our study, when facing the opportunity to donate money, the more generous participants showed increased activation in dorsomedial prefrontal cortex (DMPFC), which, together with temporoparietal junction (TPJ) and precuneus forms a social cognition network that is involved in forming impressions of others and in thinking about the needs, goals, and beliefs of others (Frith and Frith, 1999; Van Overwalle, 2009). Thus, altruistic behavior may be related to a more thorough evaluation of the needs and goals of others rather than one's own needs. Consistent with this notion, another donation study observed that activation in right TPJ was correlated with the participants' willingness to donate money to a charitable organization (Hare et al., 2010). Furthermore, neuroanatomical differences in gray matter volume in TPJ have been shown to be strongly associated with altruistic behavior (Morishima et al., 2012), providing a potential biological substrate that may underlie the stability of altruistic choices.

Taken together, the findings reviewed here suggest that core values may indeed exert their effects on decisions and behaviors via modulations of the neural regions involved in the computation of economic value: Participants with a value hierarchy dominated by self-centered core values make more selfish decisions and show a concurrent stronger activation of the ventral striatum (Brosch et al., 2011). Thus, participants with self-centered core values may perceive selfish choices and behaviors as more rewarding, and as a consequence will show these behaviors more often than participants with less self-centered core values. Altruistic behaviors may also be reflected in differential activation of the ventral striatum (Moll et al., 2006; Harbaugh et al., 2007), but may additionally involve an increased recruitment of social cognition regions such as DMPFC (Brosch et al., 2011) and TPJ (Hare et al., 2010), which are involved in perspective-taking and thinking about the needs and goals of others. During charitable choices, social cognition regions show increased connectivity with regions representing economic value (Hare et al., 2010), and may thus increase the expected economic reward value of selfless actions.

Thus, when a person with a given hierarchy of core values faces a concrete decision situation, these core values may exert their influence on individual choices and behaviors by directly modulating the computations of the expected reward value for the different options. Previous theorizing in social psychology and sociology has conceptualized the link between core value and behavior as relatively indirect, by postulating that core values impact on the beliefs and norms of an individual which then result in behavioral differences (Dietz et al., 2005) or by assuming that value-congruent behavior is mainly driven by an individual's need for consistency between one's beliefs and actions (Rokeach, 1973). We propose that, in addition to these indirect pathways, a more direct path may underlie the impact of core value on behavior. By modulating the economic value computations for different behavioral options, core values may directly impact on the perceived reward value of the different behavioral options (see also Feather, 1995). Of course, it must be noted that all neuroimaging studies cited here have used financial decisions, and have linked core value related decision-making to higher sensitivity to monetary reward only. There are many different types of rewards, including primary rewards such as food or erotic stimuli, as well as secondary rewards such as money or power. It remains to be shown that the findings reviewed here can generalize to other situations and types of rewards. A recent meta-analysis (Sescousse et al., 2013) confirmed that the neural network computing reward value is similarly activated by different kinds of primary and secondary rewards. However, it would be highly interesting to investigate individual differences in sensitivity to different types of rewards as a function of the individual core value hierarchy (e.g., comparing the reward value of erotic stimuli in participants with highly conservative values vs. participants with pronounced stimulation and hedonism values)^1^.

In addition to this direct impact of core values on neural representations of economic value in the striatum and VMPFC, as well as their modulations via social cognition regions such as TPJ and DMPFC, a more indirect pathway by which core values impact on individual beliefs and norms may play an important role: Core values form an important part of our self-concept, i.e., they help us define ourselves. Thinking about oneself as “a person who values benevolence” represents a motivationally important long-term goal that may promote core value-congruent behaviors even in the absence of concrete choice situations or rewarding options. For example, a person who values benevolence may frequently make efforts to select situations and environments in which concrete altruistic behaviors can be realized, such as going to fund-raisers or charity sales, in order to act accordingly to his beliefs.

The findings reviewed here furthermore suggest a new perspective on the mechanisms that may underlie the development of differences in individual (or cultural) core value hierarchies: Some groups or individuals may habitually show stronger sensitivity of economic value regions when receiving valued objects, which may be due to either genetic factors or epigenetic factors such as social reinforcement. Habitually stronger reward sensitivity may lead to an increase in self-interested behavior via positive reinforcement and to a more positive evaluation of prospective outcomes of such a behavior in related decision-making processes. This may result in an increased probability of choosing selfish alternatives. Similar to the role of self-perception in attitude formation (Bem, 1972), habitual choice of selfish behaviors may crystallize in an accordingly adjusted core value hierarchy that emphasizes self-centered values. Once these values become integral part of the self-concept, the explicit representation of the importance of certain classes of behavior may furthermore drive decisions and behaviors, by combining explicit and implicit reinforcing mechanisms. The model outlined in this paper should be considered as a starting point only, as research on the neural correlates and mechanisms of core values is at an early stage. We hope, however, that our contribution will stimulate further research that focuses on the role of individual differences in decision-making and the underlying neural mechanisms. In this context, economists recently have begun investigating the impact of individual differences in personality traits (e.g., “Big Five”) on economic decision-making (Rustichini, 2009), suggesting, for example, that neuroticism is linked to a lower willingness to accept risks, and extraversion to a reduced aversion to ambiguity. Somewhat related to the egoism-altruism dimension discussed in the present paper, it has been suggested that the personality dimension of Agreeableness may be related to higher cooperation with others. Initial data from a Prisoner's Dilemma game seems to support this link Rustichini et al. (2011). Future research on core values should aim at measuring personality dimensions and the individual core value hierarchy simultaneously, to assess which constructs are more powerful predictors of individual decisions and behaviors.

Whereas the model outlined here focuses on the core value dimension “self-enhancement vs. self-transcendence,” the model by Schwartz (1992) contains a second dimension, labeled “openness to change vs. conservation.” While there is hardly any neuroimaging research directly investigating this core value dimension, a number of studies have investigated neural correlates of political liberalism vs. conservatism (Jost and Amodio, 2012), a dimension that can plausibly be related to the core value dimension. Interestingly, these findings suggest that political conservatism is associated with more persistence-related errors and reduced neural responses of an error-detection system centered on anterior cingulate cortex (ACC) during a Go/No-Go task (Amodio et al., 2007). Similar results have been observed for highly religious individuals (Inzlicht et al., 2009). Whereas in these studies differential neurocognitive effects are found after a decision/behavior, when the consequences of the decision are assessed and errors are detected, it remains to be explored whether “openness to change vs. conservation”-core values may also be related to a differential weighing of the perceived economic value of different options before a decision is made.

Taken together, in this contribution we aimed at demonstrating the feasibility and usefulness of an integration of economic value research and core value research. We have suggested potential mechanisms by which core values, explicitly represented as long-term goals anchored in the self-schema, may drive concrete decisions and behaviors by acting on neural regions representing economic value. Core value research provides a theoretically elaborate and empirically validated concept that allows predicting and explaining individual differences in value-based decision-making. The theoretical integration of the different concepts opens up several new and exciting topics of research, some of them with the potential for considerable societal impact. For instance, the links between core values and behavior are sometimes relatively weak (Bardi and Schwartz, 2003). As an example, many people claim that for them the protection of the environment is an important value, but do not show consistent environmentally friendly behavior (Dietz et al., 2005). Neuroimaging research may contribute to developing targeted interventions that aim at increasing the effect of environmental core values on the corresponding behavior by exploring how situations need to be framed to elicit a high economic value of the desired behavior. Many other examples are possible. We hope that the ideas outlined here will be valuable for many researchers who care about value, and will stimulate further integration of the different value literatures.

### Conflict of interest statement

The authors declare that the research was conducted in the absence of any commercial or financial relationships that could be construed as a potential conflict of interest.

## Footnotes

^1^We thank one of our reviewers for providing this example.
